# Validation of AI-assisted ThinPrep® Pap test screening using the Genius^TM^ Digital Diagnostics System

**DOI:** 10.1016/j.jpi.2024.100391

**Published:** 2024-07-02

**Authors:** Richard L. Cantley, Xin Jing, Brian Smola, Wei Hao, Sarah Harrington, Liron Pantanowitz

**Affiliations:** a Department of Pathology, University of Michigan-Michigan Medicine, 2800 Plymouth Rd, Building 35, Ann Arbor, MI 48109, USA; b Department of Biostatistics, University of Michigan School of Public Health, 1415 Washington Heights, Ann Arbor, MI 48109, USA; c Scientific Affairs, Hologic, Inc., 250 Campus Drive, Marlborough, MA 01752, USA; d Department of Pathology, University of Pittsburgh Medical Center, 5230 Centre Avenue, Pittsburgh, PA 15232, USA

**Keywords:** Artificial intelligence, Cytology, Digital pathology, Pap test, Screening, Whole-slide imaging

## Abstract

Advances in whole-slide imaging and artificial intelligence present opportunities for improvement in Pap test screening. To date, there have been limited studies published regarding how best to validate newer AI-based digital systems for screening Pap tests in clinical practice. In this study, we validated the Genius™ Digital Diagnostics System (Hologic) by comparing the performance to traditional manual light microscopic diagnosis of ThinPrep**®** Pap test slides. A total of 319 ThinPrep**®** Pap test cases were prospectively assessed by six cytologists and three cytopathologists by light microscopy and digital evaluation and the results compared to the original ground truth Pap test diagnosis. Concordance with the original diagnosis was significantly different by digital and manual light microscopy review when comparing across: (i) exact Bethesda System diagnostic categories (62.1% vs 55.8%, respectively, *p* = 0.014), (ii) condensed diagnostic categories (76.8% vs 71.5%, respectively, *p* = 0.027), and (iii) condensed diagnoses based on clinical management (71.5% vs 65.2%, respectively, *p* = 0.017). Time to evaluate cases was shorter for digital (M = 3.2 min, SD = 2.2) compared to manual (M = 5.9 min, SD = 3.1) review (t(352) = 19.44, *p* < 0.001, Cohen's d = 1.035, 95% CI [0.905, 1.164]). Not only did our validation study demonstrate that AI-based digital Pap test evaluation had improved diagnostic accuracy and reduced screening time compared to light microscopy, but that participants reported a positive experience using this system.

## Highlights

• The GeniusTM Digital Diagnostics System represents the first Food and Drug Administration-approved system for screening of Pap test cytology.
• We performed a prospective validation study directly comparing AI-assisted digital review of Pap test cytology with the Genius System to light microscopy.
• Digital evaluation was non-inferior to microscopic evaluation, and in fact had higher agreement with ground-truth diagnosis, though the difference did not reach statistical significance.
• Mean evaluation time was lower for the digital evaluation compared to manual light microscopy evaluation, and participants reported a positive experience using the system.

## Introduction

Cervical cancer is the fourth most common cancer among women worldwide, with approximately 660,000 new cases and 350,000 deaths annually.^1^ In the United States, approximately 13,820 new cases and 4360 deaths occur annually due to cervical cancer.^2^ The Pap test for cervical cancer screening was first proposed by Dr. George Papanicolaou in 1928, and by the 1940s had demonstrated its efficacy in diagnosing cancers and pre-cancerous lesions of the uterine cervix.^3^ By the 1960s, the Pap test was adopted for routine practice throughout the United States and has been the gold-standard for cervical cancer screening. In the United States, the incidence of cervical cancer further dropped over 80% after the introduction and adoption of the Pap test.^4^ Today, millions of Pap tests are accordingly performed annually in the United States.^5^

The 1990s saw the advent of liquid-based cytology (LBC) preparations, with the first being Hologic's ThinPrep® system. Though LBC has advantages over conventional smears for light microscopic examination, LBC technology was developed with the primary goal of optimizing cytology specimens for automated computer-assisted Pap test screening.^5^ In the 2000s, automated LBC screening devices came to market; the first Food and Drug Administration (FDA)-approved system for screening Pap tests was the ThinPrep Imaging System (TIS) which identifies field of view (FOV) with the most concerning cells.^6^^,^^7^ Even with the assistance of screening systems such as TIS, manual review of abnormal cases using a light microscope is still generally required. However, manual cytology evaluation is subjective. Hence, sensitivity rates vary among laboratories, and intra- and interobserver variability is significant, especially for indeterminate diagnoses such as atypical squamous cells (ASCs) and atypical glandular cells (AGCs).^8^, ^9^, ^10^, ^11^, ^12^, ^13^, ^14^ Moreover, screening Pap tests by employing a light microscope is labor-intensive, requires highly skilled cytologists, and relies on a physical glass slide that limits portability.

**Fig. 1:**
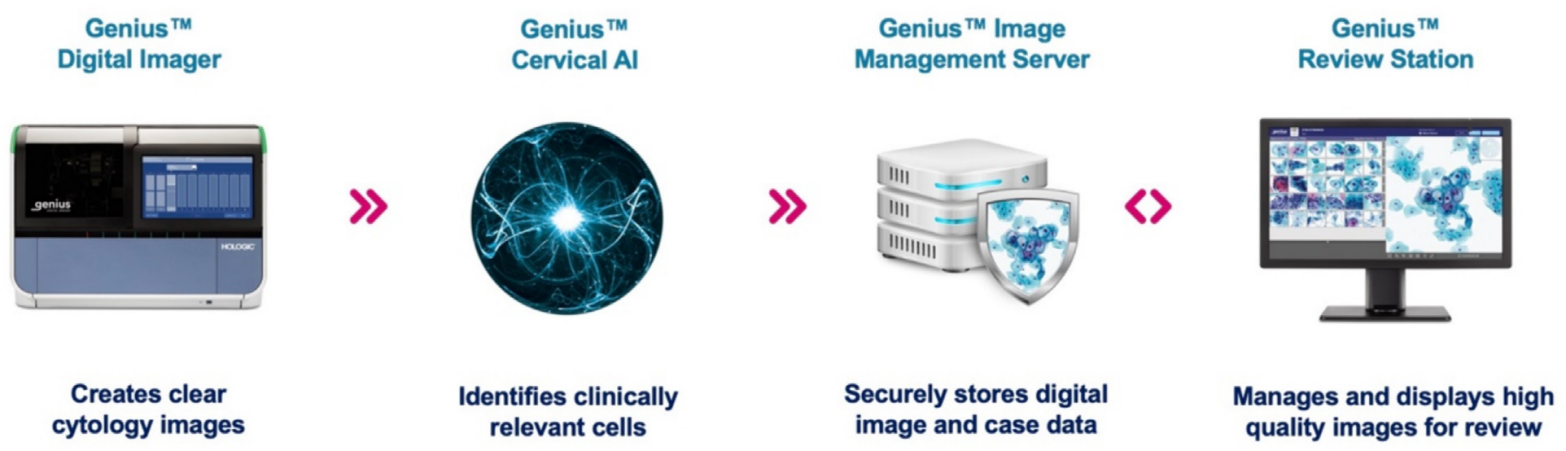
The Genius™ Digital Diagnostic System is comprised of a whole-slide scanner, server, image management software, deep learning-based AI algorithm, and review station.

Advances in artificial intelligence (AI), computer processing, and whole-slide imaging (e.g., volumetric scanning) have allowed further development of automated Pap test screening in recent years. Hologic developed a digital cytology system that leverages these technological advances for computer-assisted Pap test screening known as the Genius Digital Diagnostic System.^15^ This Genius system includes a digital imager, image management server, and a review station (Fig. 1).^16^ The digital imager scans ThinPrep slides in multiple Z planes, allowing in-focus imaging of multiple planes within the same image file (i.e., volumetric scanning), with scanning taking approximately 1 min per slide.^6^ A deep learning-based AI algorithm (“Genius Cervical AI algorithm”) identifies areas of interest in the digitized slide, and the most clinically relevant image patches are presented in a gallery of images for review on a dedicated workstation running the review software. Unlike the previous TIS system, no light microscopy slide review is required. The Genius Digital Diagnostic System recently received FDA-clearance for cervical cancer screening in the United States.

The use of whole-slide imaging coupled with AI in Pap test cytology has the potential to significantly impact cytology practice. To date, there have been limited studies published regarding how best to validate newer AI-based digital systems for screening Pap tests in clinical practice.^17^^,^^18^ In this study, we share our experience validating the Genius Digital Diagnostics System by comparing its performance to traditional manual light microscopic diagnosis of ThinPrep Pap test slides. In addition, we evaluated the potential impact of digital slide review on turnaround time to determine the overall efficiency of digital review compared to manual light microscopy.

## Methods and materials

Institutional Review Board approval was obtained before the commencement of this study.

### Training and case selection

**Fig. 2:**
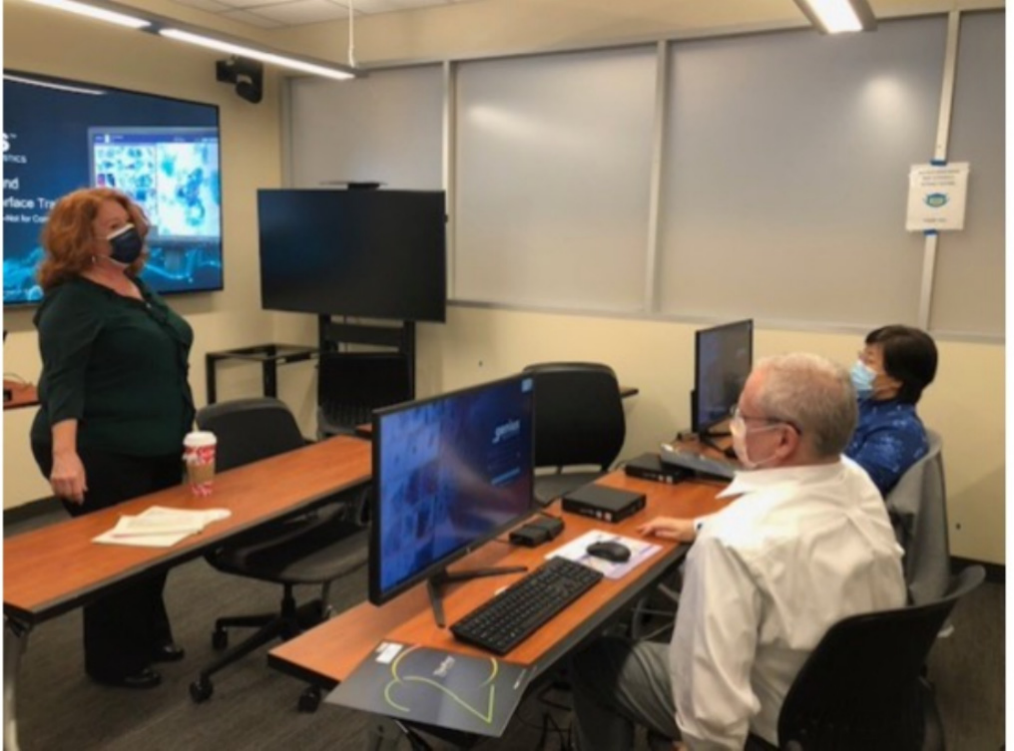
In-person training of participants at the University of Michigan to use Hologic's Genius™ Digital Diagnostics system.

There were six cytologists (cytotechnologists = CTs) and three board-certified cytopathologists (CPs) who voluntarily participated in the study. Before reviewing study cases, representatives from Hologic trained participants in the use of the Genius Digital Diagnostics system over the course of 1.5 days (Fig. 2). Participants demonstrated competence in the system by testing on three unknown slide sets.

Our cytology archives were searched to identify 320 ThinPrep Pap test cases recently signed out from adult patients. To ensure adequate representation of all categories from the Bethesda System (TBS) typically encountered in our routine clinical practice, we collected 11 unsatisfactory specimens, 153 negative for intraepithelial lesion or malignancy (NILM), 48 atypical squamous cells of undetermined significance (ASCUS), 32 low-grade squamous intraepithelial lesions (LSIL), 22 atypical squamous cells, cannot exclude HSIL (ASC—H), 33 high-grade squamous intraepithelial lesions (HSIL), 18 AGCs, and three malignant cases (one adenocarcinoma and two squamous cell carcinomas). However, one case with an original assessment of LSIL did not have a digital assessment, and was therefore excluded from analysis, leaving a total of 319 cases for analysis. Data captured included the original Pap test diagnosis, which was considered the “ground-truth” diagnosis for this study, as well as HPV test results for atypical cases if applicable, patient age, and clinical history with last menstrual period when available. Patient age ranged from 19 to 77 years (*M* = 33.6 years, Mdn = 29 years).

### Data collection

**Fig. 3:**
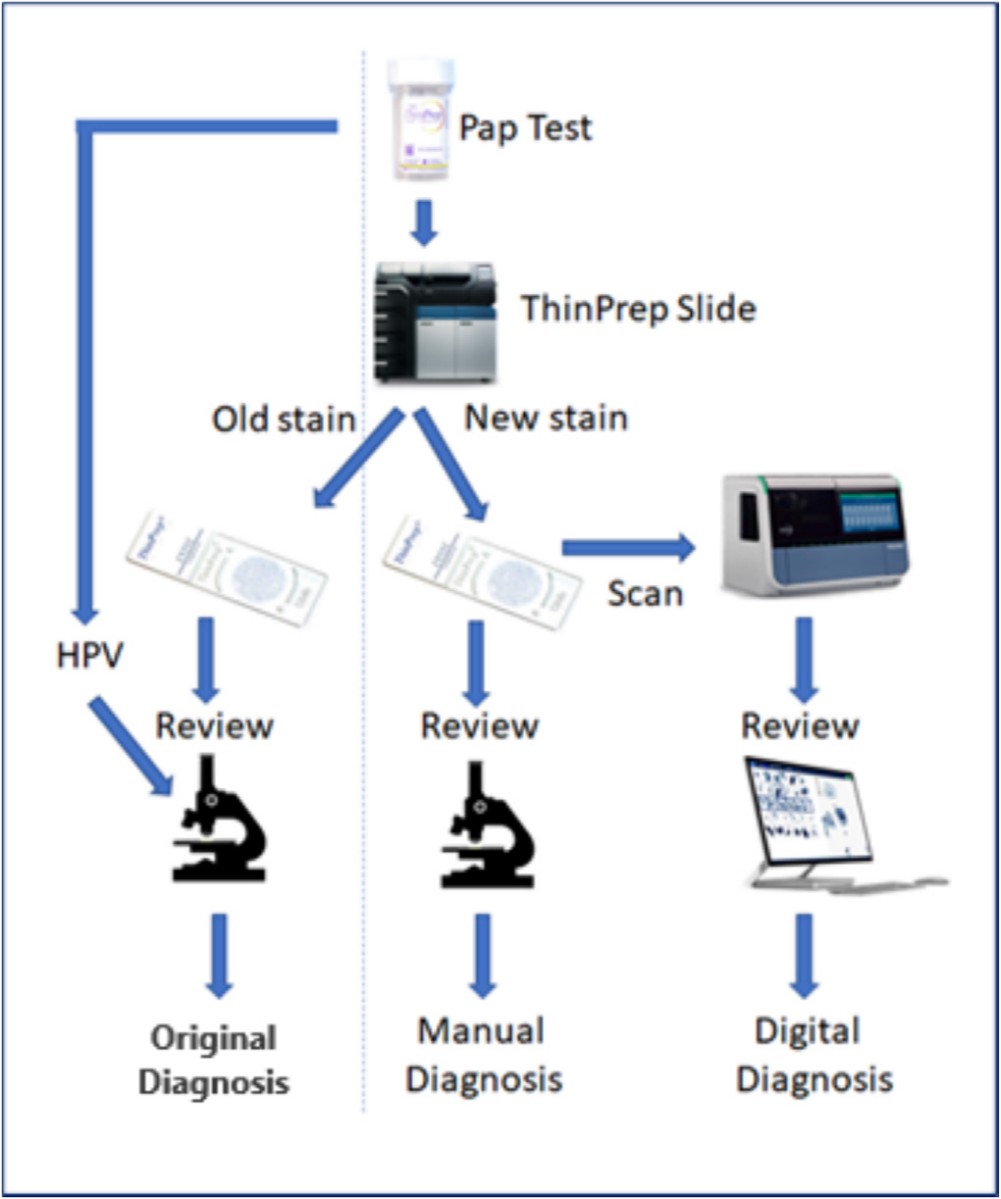
Validation study outline. After ThinPrep Pap tests were collected for traditional light microscopy and HPV testing where applicable, residual fluid was retained to produce a new ThinPrep slide for scanning with the Genius whole-slide imager. The new slides were then evaluated prospectively by both light microscopy and AI-assisted digital review by all participants, with a two-week washout period between manual and digital review.

**Fig. 4:**
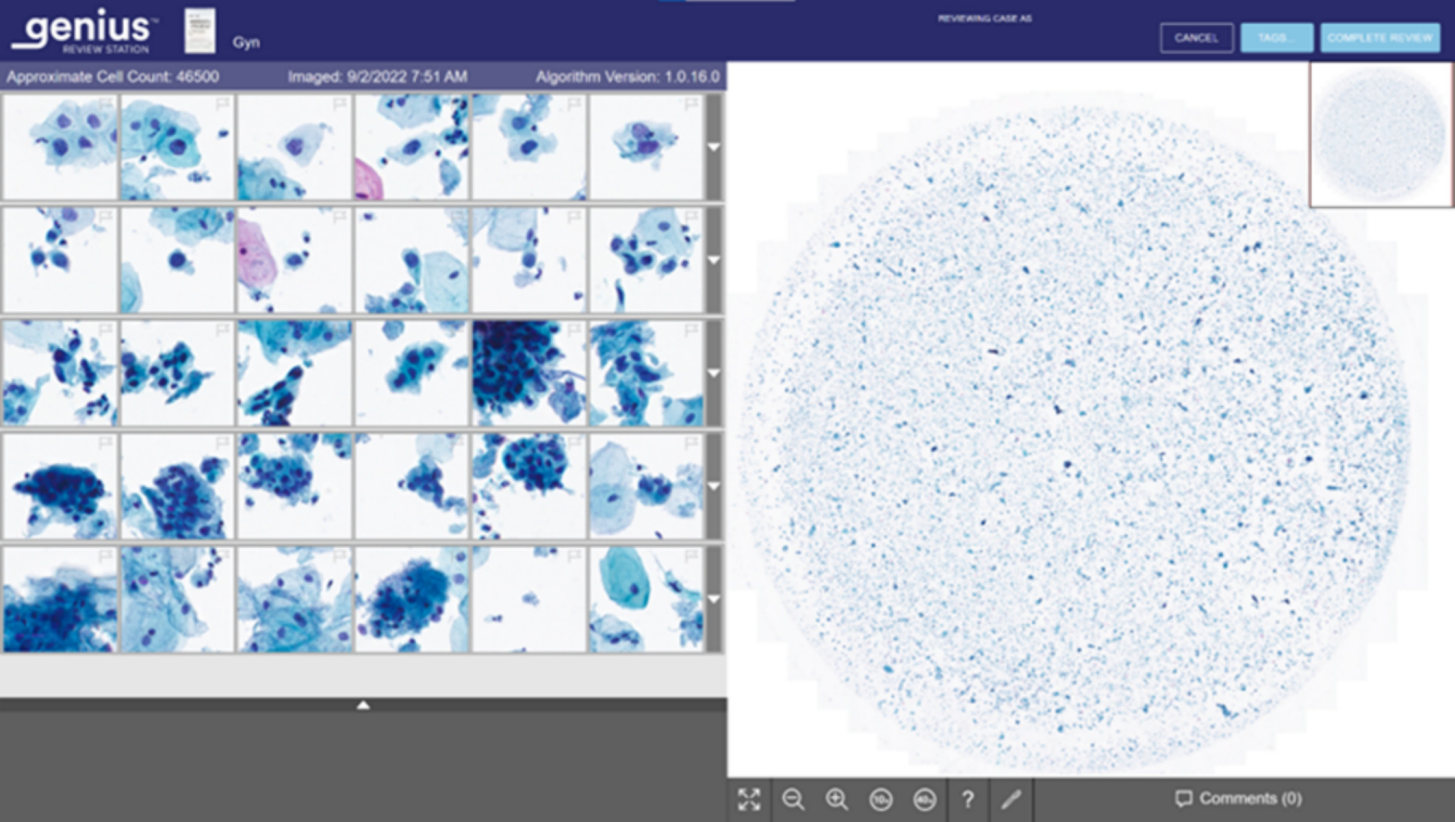
Screenshot of the Genius Review Station. The left gallery displays 30 images that are selected for view by the Genius Cervical AI algorithm. The gallery is expandable to 60 images. The entire ThinPrep cell spot is also available on the right half of the monitor for the reviewer to examine.

Residual ThinPrep fluid that would ordinarily be discarded was used to produce new ThinPrep slides, stained using the TIS slide staining protocol, according to manufacturer specifications for the Genius system. The new slides were scanned via whole-slide imaging on the Genius Digital Imager, and the new glass slides were also retained (Fig. 3). Each case was prospectively reviewed by light microscopy and digital interface (Fig. 4), with at least a two-week “washout” period between light microscopic and digital evaluation. Participants were blinded to the original Pap test diagnosis and HPV status. To simulate typical pathology practice, cases were initially evaluated by CTs. NILM cases were “signed out” by the CT, whereas all atypical or reactive cases were then evaluated by a CP. The final diagnosis was recorded along with time (in minutes) taken to reach a diagnosis.

### Light microscopy versus digital evaluation

For each patient, the final diagnosis was based on CP diagnosis if available, or CT diagnosis if no CP evaluation was performed (i.e., non-reactive NILM cases). To evaluate accuracy of diagnosis with ground truth, manual light microscopy versus digital results were compared. Diagnostic assessments based on a simplified TBS classification included eight categories (unsatisfactory, NILM, ASCUS, LSIL, ASC—H, HSIL, AGC, and malignant). Two condensed classifications were also used. In one, all diagnoses of ASCUS+ were grouped, resulting in a 3-category classification (unsatisfactory, NILM, ASCUS+). The second condensed classification was based on clinical management, with ASCUS and LSIL combined, and ASC—H, HSIL, AGC, and malignant diagnoses combined, resulting in a 4-category classification (unsatisfactory, NILM, ASCUS/LSIL, ASC-H/HSIL/AGC/malignant). Accuracy assessments were based upon a diagnostic match to the original Pap test result (ground truth) and were calculated for the 8-category diagnostic range as well as the condensed 3- and 4-category groups. A McNemar test was used to compare the concordance between the ground truth and manual light microscopy versus digital review. Statistical significance was achieved with a *p*-value less than 0.05.

Diagnostic agreement between the manual light microscopy review and AI-assisted digital review was calculated for the 8-category classification (unsatisfactory, NILM, ASCUS, LSIL, ASC—H, HSIL, AGC, and malignant), 4-category classification (unsatisfactory, NILM, ASCUS/LSIL, ASC-H/HSIL/AGC/malignant), and 3-category classification (unsatisfactory, NILM, ASCUS+).

HPV status was explored for cases that were signed out as ASCUS or ASC—H, and HPV results were recorded as negative (HPV-) or positive (HPV+) if the test was performed. Among the 70 cases with an ASCUS or ASC-H diagnosis, 29 had HPV- results, 26 had HPV+ results, and 15 did not have HPV testing. For cases where HPV results were known (negative or positive), the diagnostic concordance was compared for digital and manual light microscopy to the ground-truth diagnosis (ASCUS or ASC—H) for HPV- and HPV+. Concordance was considered an exact match to the ground truth (ASCUS or ASC—H). A McNemar test was used to compare the concordance between the ground truth and manual light microscopy versus digital review.

Efficiency was calculated based on time spent in minutes on manual light microscopy versus digital review. Participants recorded the amount of time spent evaluating each case, and the total case time was compared by review method. A paired samples *t*-test was used to compare the total time spent for manual light microscopy versus digital assessment. Cytologist review time was not recorded for 18 manual light microscopy reviews and 29 digital reviews, and therefore the remaining 272 pairs of times were compared. Due to either NILM diagnosis or undocumented CP manual light microscopy or digital time, 81 pairs of times were compared for CPs. Aggregate time for manual light microscopy and digital reviews were based on the 272 cytologist pairs and 81 CP pairs.

## Results

### Diagnostic accuracy

Manual light microscopy and digital diagnoses were compared against the ground truth (original Pap test diagnosis). Digital evaluation matched the 8-category diagnostic classification in 198 cases (62.1%), whereas manual light microscopy evaluation diagnosis matched in 178 (55.8%) cases (Table 1). Digital evaluation showed greater agreement with ground-truth diagnosis for nearly every diagnostic category (NILM, ASCUS, LSIL, AGC, HSIL). The most common reason for manual review discordance were *n* = 29 cases that were assessed as NILM on manual light microscopy review, with a ground-truth assessment of ASCUS (Table 2). Similarly, the most common reason for digital discordance were *n* = 26 cases that were assessed as NILM on digital review, with a ground-truth assessment of ASCUS (Table 3).

**Table 1:** Comparison of concordance for manual light microscopy versus digital evaluation to ground-truth diagnosis (based on the Bethesda classification, simplified to eight categories).

	Ground truth (signed-out Pap diagnosis)	Manual concordance with ground truth		Digital concordance with ground truth	
	*n*	*n*	%	*n*	%
Unsatisfactory	11	10	90.9%	9	81.8%
NILM	153	115	75.2%	128	83.7%
ASCUS	48	7	14.6%	11	22.9%
LSIL	31	21	67.7%	23	74.2%
ASC-H	22	3	13.6%	3	13.6%
AGC	18	3	16.7%	4	22.2%
HSIL	33	17	51.5%	18	54.5%
Malignant	3	2	66.7%	2	66.7%
Total	319	178	55.8%	198	62.1%

**Table 2:** Manual light microscopy review assessment versus ground-truth diagnoses.

		Manual light microscopy review								
		Unsat	NILM	ASCUS	LSIL	ASC-H	AGC	HSIL	Malignant	Total
Ground truth	Unsat	10	0	0	0	0	1	0	0	11
	NILM	9	115	9	6	7	5	2	0	153
	ASCUS	1	29	7	8	0	3	0	0	48
	LSIL	0	2	4	21	2	0	2	0	31
	ASC-H	0	6	3	1	3	2	6	1	22
	AGC	3	7	1	0	0	3	2	2	18
	HSIL	0	4	1	7	2	0	17	2	33
	Malignant	0	0	0	0	0	1	0	2	3
	Total	23	163	25	43	14	15	29	7	319

**Table 3:** AI-assisted digital review assessment versus ground-truth diagnoses.

		AI-assisted digital review								
		Unsat	NILM	ASCUS	LSIL	ASC-H	AGC	HSIL	Malignant	Total
Ground truth	Unsat	9	2	0	0	0	0	0	0	11
	NILM	8	128	11	5	0	0	1	0	153
	ASCUS	2	26	11	5	3	1	0	0	48
	LSIL	1	0	5	23	1	0	1	0	31
	ASC-H	0	4	3	1	3	1	10	0	22
	AGC	4	7	0	0	1	4	1	1	18
	HSIL	0	3	3	4	4	0	18	1	33
	Malignant	0	0	0	0	0	1	0	2	3
	Total	24	170	33	38	12	7	31	4	319

When considering concordance based on the 8-category diagnostic classification, digital evaluation more commonly concurred with the ground-truth diagnosis (198 concordant cases, 62.1%) compared to light microscopy review assessments (178 concordant cases, 55.8%), *p* = 0.014 (Table 4). Cytologist digital reviews had a higher concordant rate (197, 61.8%) than cytologist light microscopy reviews (178, 55.8%), *p* = 0.024. Similarly, CP digital reviews had a higher concordant rate (63, 52.5%) than CP light microscopy reviews (50, 41.7%), *p* = 0.012.

**Table 4:** Concordance of manual light microscopy and digital assessments with ground-truth diagnosis (based on the Bethesda classification, simplified to eight categories). Comparing both screening methods with the original diagnosis, there was greater concordance with digital (62.1%) than manual light microscopy (55.8%).

		Digital		
		Concordant with ground truth	Discordant with ground truth	Total
Manual light microscopy	Concordant with ground truth	155	23	178 (55.8%)
	Discordant with ground truth	43	98	141 (44.2%)
	Total	198 (62.1%)	121 (37.9%)	319

Condensing all ASCUS+ diagnoses (ASCUS, LSIL, ASC—H, HSIL, AGC, malignant), the overall concordance for both review methods by diagnostic category is shown in Table 5. Using this condensed 3-category classification (unsatisfactory, NILM, ASCUS+), concordance with review method and the original diagnosis was statistically significantly different (245 concordant digital cases (76.8%) and 228 concordant manual light microscopy cases (71.5%), *p* = 0.027 (Table 6)). There was not a significant difference between concordance with ground truth and cytologist manual light microscopy review (232, 72.7%) versus cytologist digital review (241, 75.5%), *p* = 0.199. There was a significant difference between concordance with ground truth and CP manual light microscopy review (98, 81.7%) versus CP digital review (106, 88.3%), *p* = 0.033.

**Table 5:** Comparison of concordance of manual light microscopy versus digital evaluation to condensed ground-truth diagnosis (condensed all ASCUS+).

	Ground truth (signed-out Pap diagnosis)	Manual concordance with ground truth		Digital concordance with ground truth	
	*n*	*n*	%	*n*	%
Unsatisfactory	11	10	90.9%	9	81.8%
NILM	153	115	75.2%	128	83.7%
ASCUS+	155	103	66.5%	108	69.7%
Total	319	196	61.4%	212	66.5%

**Table 6:** Concordance of manual light microscopy and digital assessments with condensed ground-truth diagnosis (condensed all ASCUS+). Comparing both screening methods with the original diagnosis, there was greater concordance with digital (76.8%) than manual light microscopy (71.5%).

		Digital		
		Concordant with ground truth	Discordant with ground truth	Total
Manual light microscopy	Concordant with ground truth	207	21	228 (71.5%)
	Discordant with ground truth	38	53	91 (28.5%)
	Total	245 (76.8%)	74 (23.2%)	319

Overall concordance when the diagnostic categories are grouped based on clinical management (combined ASCUS and LSIL, combined ASC—H, HSIL, AGC, and malignant diagnoses) is shown in Table 7. Using this condensed 4-category classification based on clinical management (unsatisfactory, NILM, ASCUS/LSIL, ASC-H/HSIL/AGC/malignant), concordance with review method and the original diagnosis was statistically significantly different (208 concordant manual light microscopy cases (65.2%) and 228 concordant digital cases (71.5%), *p* = 0.017 (Table 8). There was a significant difference between concordance with ground truth and cytologist manual light microscopy review (206, 64.6%) versus cytologist digital review (223, 69.9%), *p* = 0.032. There was also a significant difference between concordance with ground truth and CP manual light microscopy review (79, 65.8%) versus CP digital review (90, 75.0%), *p* = 0.034.

**Table 7:** Comparison of concordance of manual light microscopy versus digital evaluation to condensed ground-truth diagnosis (grouped ASCUS and LSIL, grouped ASC—H, HSIL, AGC, malignant).

	Ground truth (signed-out Pap diagnosis)	Manual concordance with ground truth		Digital concordance with ground truth	
	*n*	*n*	%	*n*	%
Unsatisfactory	11	10	90.9%	9	81.8%
NILM	153	115	75.2%	128	83.7%
ASCUS/LSIL	79	40	50.6%	44	55.7%
ASC-H/HSIL/AGC/Malignant	76	43	56.6%	47	61.8%
Total	319	196	61.4%	212	66.5%

**Table 8:** Concordance of manual light microscopy and digital assessments with condensed ground-truth diagnosis (grouped ASCUS and LSIL, grouped ASC—H, HSIL, AGC, malignant). Comparing both screening methods with the original diagnosis, there was greater concordance with digital (71.5%) than manual light microscopy (65.2%).

		Digital		
		Concordant with ground truth	Discordant with ground truth	Total
Manual light microscopy	Concordant with ground truth	183	25	208 (65.2%)
	Discordant with ground truth	45	66	111 (34.8%)
	Total	228 (71.5%)	91 (28.5%)	319

### Agreement among review methods

There were 220 cases (69.0%) with an exact diagnostic category match between light microscopy and AI-assisted digital review (Table 9). When condensed to a 3-category classification (combined ASCUS+ diagnoses), the agreement increased to *n* = 257 (80.6%) of cases (Table 10). The 4-category classification based on clinical management had an agreement among *n* = 237 cases (74.3%), shown in Table 11.

**Table 9:** Diagnostic agreement between manual light microscopy review assessment and AI-assisted digital review.

		AI-assisted digital review								
		Unsat	NILM	ASCUS	LSIL	ASC-H	AGC	HSIL	Malignant	Total
Manual light micro-scopy review	Unsat	18	5	0	0	0	0	0	0	23
	NILM	3	137	15	3	3	2	0	0	163
	ASCUS	0	11	8	2	1	0	3	0	25
	LSIL	1	3	5	28	3	0	3	0	43
	ASC-H	1	6	1	2	1	0	3	0	14
	AGC	1	6	2	0	1	4	1	0	15
	HSIL	0	2	2	3	2	0	20	0	29
	Malignant	0	0	0	0	1	1	1	4	7
	Total	24	170	33	38	12	7	31	4	319

**Table 10:** Diagnostic agreement between manual light microscopy review assessment and AI-assisted digital review (condensed all ASCUS+).

		AI-assisted digital review			
		Unsat	NILM	ASCUS+	Total
Manual light microscopy review	Unsat	18	5	0	23
	NILM	3	137	23	163
	ASCUS+	3	28	102	133
	Total	24	170	125	319

**Table 11:** Diagnostic agreement between manual light microscopy review assessment and AI-assisted digital review (grouped ASCUS and LSIL, grouped ASC—H, HSIL, AGC, malignant).

	AI-assisted digital review					
	Unsat	NILM	ASCUS/LSIL	ASC-H/HSIL/AGC/Malignant	Total	
Manual light micro-scopy review	Unsat	18	5	0	0	23
	NILM	3	137	18	5	163
	ASCUS/LSIL	1	14	43	10	68
	ASC-H/HSIL/AGC/Malignant	2	14	10	39	65
	Total	24	170	71	54	319

### Atypical squamous cells and HPV status

Among the 55 ASC cases with known HPV results, 26 (47.3%) were HPV+ and 29 (52.7%) were HPV-. There was no significant difference in concordance with ground truth for manual light microscopy and digital evaluation for either HPV- (Table 12) or HPV+ (Table 13) ASC Pap tests. For HPV+ ASC cases, concordance with ground truth occurred with manual light microscopy evaluation in three (11.5%) cases versus four (15.4%) of digital cases (*p* = 0.655). For HPV- ASC cases, concordance with ground truth occurred with manual light microscopy evaluation in four (13.8%) cases versus six (20.7%) of digital cases (*p* = 0.480).

**Table 12:** For cases with an original diagnosis of ASCUS or ASC-H and HPV-negative result, concordance is shown for manual light microscopy and digital assessments with ground truth (*p*-value of 0.480).

		Digital		
		Concordant with ground truth	Discordant with ground truth	Total
Manual light microscopy	Concordant with ground truth	1	3	4 (13.8%)
	Discordant with ground truth	5	20	25 (86.2%)
	Total	6 (20.7%)	23 (79.3%)	29

**Table 13:** For cases with an original diagnosis of ASCUS or ASC-H and HPV-positive result, concordance is shown for manual light microscopy and digital assessments with ground truth (*p*-value of 0.655).

		Digital		
		Concordant with ground truth	Discordant with ground truth	Total
Manual light microscopy	Concordant with ground truth	1	2	3 (11.5%)
	Discordant with ground truth	3	20	23 (88.5%)
	Total	4 (15.4%)	22 (84.6%)	26

### Turnaround times

**Fig. 5:**
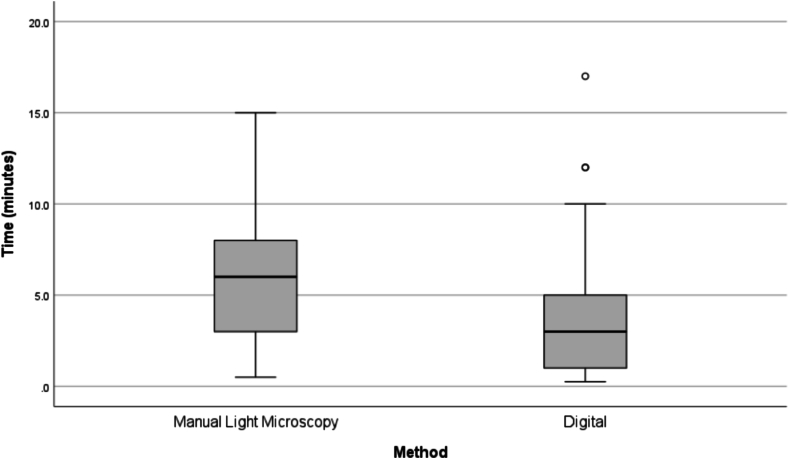
Time for diagnostic assessment of Pap test specimen by review method (manual light microscopy versus digital) for aggregate cytologist and CP reviews. Based on paired samples, digital review was faster than manual light microscopy review.

The mean evaluation time for manual light microscopy slide review among the aggregate group of cytologists and CPs was 5.9 ± 3.1 min, compared to 3.2 ± 2.2 min for digital evaluation (Fig. 5). The difference was statistically significant (t(352) = 19.44, *p* < 0.001, Cohen's d = 1.035, 95% CI [0.905, 1.164]), confirming that digital evaluation was significantly less time-consuming overall. Among the cytologists alone, the average digital review time (*M* = 3.8 min, SD = 2.1) was statistically significantly shorter than manual light microscopy review time (*M* = 7.1 min, SD = 2.6), *t*(271) = 19.80, *p* < 0.001, Cohen's d = 1.201, 95% CI [1.044, 1.356]. Among the CPs, the average digital review time (*M* = 1.2 min, SD = 1.0) was statistically significantly shorter than manual light microscopy review time (*M* = 2.2 min, SD = 1.3), *t*(80) = 6.67, *p* < 0.001, Cohen's d = 0.741, 95% CI [0.493, 0.985].

### Subjective assessment

All participants of the validation study reported a positive experience using the system.

## Discussion

Over the past three decades, significant advances have been made with computer-assisted Pap test screening. The first commercially available product to offer automation assistance was the PAPNET, introduced in 1992 by Neuromedical Systems Inc. for re-review of Pap tests previously diagnosed as negative.^6^^,^^19^^,^^20^ At that time, slides had to be shipped to a central facility for scanning and processing. In 1997, PAPNET-on-Cyte was introduced, which brought the imaging and processing device on site to laboratories. However, the device did not gain market acceptance, likely in part due to the limited utility of an expensive device intended only for rescreening negative Pap tests, which yielded only minimal increases in sensitivity. AutoPap 300QC was FDA approved in 1998 for diagnosing negative conventional and 2001 for negative SurePath Pap test slides without manual review. The system was acquired by BD Diagnostics and rebranded as the BD FocalPoint Slide Profiler.^21^, ^22^, ^23^ BD later incorporated this device into its BD FocalPoint GS Imaging System, which included a robotic microscope with slide “dotting” capability to assist manual review. This device was FDA-approved for use on SurePath slides in 2008. The first widely adopted machine learning-based device intended for use in primary Pap test screening was the TIS, which was FDA-approved in 2003. The TIS analyzes cell features to identify 22 fields of view containing the cells most concerning for dysplasia. A cytotechnologist then reviews the fields on a robotic microscope which moves the slide to display the 22 fields of interest. If no abnormality is detected, the Pap test requires no additional screening. If an abnormality is detected, the slide is then fully manually screened.

More recently, with continued advances in whole-slide imaging and deep learning-based AI algorithms, several vendors have leveraged the opportunity to offer cytology laboratories commercial systems capable of fully digital Pap test review.^19^, ^20^, ^21^ While many digital systems and AI algorithms are still in development for cervical cancer screening, the Genius Digital Diagnostics System is currently the only complete system that is FDA-cleared for use in screening, diagnosis and management of cervical cytology specimens.

Fully digitizing Pap test slides introduces significant potential advantages over light microscopy and previous automated systems. “Untethering” end-users from the light microscope is a major benefit. Long-term microscope use is associated with neck and back pain, eye strain, headache, and arm injuries such as lateral epicondylosis (“tennis elbow”).^24^ Moreover, performing rapid onsite evaluation requires free movement of CTs and CPs away from their desks. This can be enabled by digital pathology, because scanned material can be viewed essentially anywhere that a digital review station is available. Full digital evaluation of cytopathology material, thus, offers both ergonomic and workflow advantages.

In this validation study, we directly compared manual light microscopy and AI-assisted digital review of Pap test cytology using the same glass slide, which was produced for the study from residual ThinPrep fluid. This was a retrospective assessment on cases previously signed out, to prevent use of active specimens which may have interfered with clinical interpretation and turnaround. However, in this manner we were able to compare the results of both manual and digital (non-inferiority) to the “ground-truth” diagnosis, which was the original Pap test diagnosis.

Although no firm guidelines are established at this time for validation of AI-assisted devices in cytology, a global survey of the American Society of Cytopathology Digital Cytology Task Force found most respondents felt 100–200 cases are an appropriate volume for clinical validation.^25^ We identified 320 cases for our study to ensure an adequate validation case volume without being overly burdensome on clinical practice. Among diagnostic cases, approximately half were negative (153) and half were ASCUS or above (155). This allowed representation of cases across the spectrum of Pap test diagnostic categories, including less common categories such as AGC and ASC—H.

Digital evaluation more often concurred with the ground truth than light microscopy for the full diagnostic category range (62.1% compared to 55.8%, respectively; *p* = 0.014), grouping ASCUS+ (76.8% compared to 71.5%, respectively; *p* = 0.027), and grouping diagnoses based on clinical management (71.5% compared to 65.2%, respectively; *p* = 0.017). In addition, the AI-assisted digital evaluation took significantly less time than manual light microscopy. This suggests that the use of the Genius Digital Diagnostic System was not only more accurate (based on matching the ground-truth diagnosis), but also more efficient (with faster review times) in our setting than manual light microscopy.

The overall moderate agreement with the ground-truth diagnosis for each review method (62.1% for digital, 55.8% for light microscopy) was not unexpected given a number of factors that may have impacted agreement. Notably, the ThinPrep slides used for this study were not the same Pap test slides on which the original diagnosis was based; rather, a new slide was rendered from residual retained fluid. The second slide was also stained to specifications for the Genius Digital Diagnostics System, so slight differences in staining compared to the original slide may have been a confounding factor. Previous investigators have noted a “learning curve” for new observers with the Pap stain protocol used with the TIS, which results in slightly darker nuclei.^22^^,^^26^ The quantity and quality of atypical cells may have varied between the ground-truth Pap test slide and the study Pap test slide. This is particularly important given the number of equivocal cases included in this study (27.6% of all cases and 53% of the abnormal cases). Moreover, participants only had had limited time (1.5 days training) to familiarize themselves with the system and this new stain. Further, although users found the digital workflow overall easy to use, it takes time to develop user trust in new technologies.

Pap tests are known to have moderate sensitivity and are subject to significant intraobserver and interobserver variability.^27^ The Bethesda Interobserver Reproducibility Studies (BIRST) found exact agreement with expert diagnosis in only 55% of cases in 2006 (BIRST-1) and in 63% of cases in 2017 (BIRST-2).^9^, ^10^, ^11^ In addition, “atypical” diagnoses are usually associated with lower reproducibility than NILM and SIL diagnoses.^11^^,^^23^ To ensure representation of all Bethesda categories, our dataset was “spiked” with 155 (48.6%) abnormal Pap tests, including 88 (27.6%) of which fell under ASCUS, ASC—H, or AGC. As in previous studies, we found lowest agreement within these atypical categories. Prior studies have also generally shown increased atypical rates with automated screening.^26^ However, in this study, there was a tendency to downgrade ASCUS to NIL for both light microscopy and digital review from ground-truth diagnosis, whereas upgrading NIL to ASCUS was less common (Table 2, Table 3).

Turnaround time was improved with use of the Genius Digital Diagnostic System compared to glass slide review, with participants generally spending a few minutes less per slide on digital review (Fig. 5), despite only having 1.5 days to familiarize themselves with the system before the study. Because this is a simulated test environment, it is hard to extrapolate the turnaround time to true clinical practice. However, it is our opinion that the adoption of this system would improve workflow efficiency based on these results. Cytotechnologists' work can involve screening dozens of slides per day. Small decreases in screening time per slide can add up quickly in this environment. It also eliminates the need for physical slide distribution, which can improve efficiency for cytotechnologists and CPs who cover multiple location sites.

To the best of our knowledge, this is the first validation study of the Genius Digital Diagnostic System comparing digital and manual evaluation of Pap tests using ThinPrep Pap test slides in the United States. Despite limited exposure to the Genius system, CPs and CTs found the digital interface user-friendly and were comfortable with the digital workflow, image resolution and case load times. In summary, AI-assisted digital evaluation in our validation study was more accurate than manual light microscopy evaluation for Pap test diagnosis, and AI-assisted digital evaluation significantly shortened time spent per case. Strengths of this study include a prospective design with direct comparison (concordance) of manual and digital evaluation of the same slide, and participation of three specialty-trained CPs and six CTs. Limitations include a modest case volume (319 Pap tests) and lack of biopsy follow-up. Nonetheless, we found that the Genius system was non-inferior to light microscopy on retrospective analysis. Overall, AI-assisted digital evaluation of whole scanned slides was a reliable and efficient method for Pap test diagnosis, with the potential to greatly improve workflow in the cytopathology laboratory and enable remote interpretation of Pap tests.

## Declaration of competing interest

The authors declare that they have no known competing financial interests or personal relationships that could have appeared to influence the work reported in this paper.
